# Biological Activity of Some Aromatic Plants and Their Metabolites, with an Emphasis on Health-Promoting Properties

**DOI:** 10.3390/molecules25112478

**Published:** 2020-05-27

**Authors:** Marek Kieliszek, Amr Edris, Anna Maria Kot, Kamil Piwowarek

**Affiliations:** 1 Department of Food Biotechnology and Microbiology, Institute of Food Sciences, Warsaw University of Life Sciences—SGGW, Nowoursynowska 159 C, 02-776 Warsaw, Poland; anna_kot@sggw.edu.pl; 2 Aroma & Flavor Chemistry Department, Food Industries & Nutrition Division, National Research Center, El Behose Street, Dokki, Cairo 12622, Egypt

**Keywords:** frankincense, myrrh, ginger, curcumin, cancer, bioactives, volatile and nonvolatile fractions

## Abstract

The biological activities of four aromatic plants, namely frankincense, myrrh, ginger, and turmeric, were reviewed in the current study. The volatile fraction (essential oil) as well as the nonvolatile fraction of these four plants showed different promising biological activities that are displayed in detail. These activities can include protection from and/or alleviation of some ailment, which is supported with different proposed mechanisms of action. This review aimed to finally help researchers to get a handle on the importance of considering these selected aromatic plants, which have not been thoroughly reviewed before, as a potential adjuvant to classical synthetic drugs to enhance their efficiency. Moreover, the results elicited in this review encourage the consumption of these medicinal plants as an integrated part of the diet to boost the body’s overall health based on scientific evidence.

## 1. Introduction

The synthetic drug era was first introduced in human history after the discovery of the “magic bullet” and synthetic antibiotics. In recent years, the world has witnessed a tremendous development of effective synthetic drugs and new generations of antibiotics that have saved the lives of millions of people. However, at present, synthetic drugs are experiencing difficulties in the treatment of some diseases caused by organisms or cells being resistant to drugs, such as multidrug-resistant bacteria and some chemotherapy-resistant tumor cells. In addition, drugs designed to cure certain ailments seem to be toxic or harmful to some other body organs. Therefore, presently, there is a strong trend to study natural sources for biologically active extracts that can be integrated with synthetic drugs for the production of more effective and safer medications. Aromatic and medicinal plants are principle candidates for this task because of the long history of the successful use of folk medicine to treat different human diseases, long before the discovery of synthetic drugs. In this regard, dozens of investigations and review articles have been published demonstrating the biological potential of aromatic plants. However, some candidates of this family, like frankincense, myrrh, ginger, and turmeric, have been reviewed to a lesser extent with less detail compared to others. Therefore, in the current review, more light will be shed on the biological potentials of these four aromatic plants to display their promising role as potential therapeutic and/or protective adjuvants to standard drugs.

## 2. Biological Activity of Frankincense (*Boswellina* spp.)

**Figure 1:**
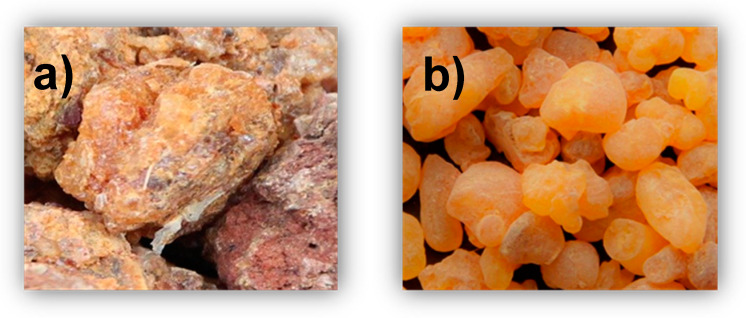
Myrrh (**a**) and frankincense (**b**).

Frankincense, also known as *Boswellia*, is a dried sap or gum resin obtained from a tree belonging to the family Burseraceae. The tree is native to the Arabian Peninsula area, northeastern Africa, and India. The gum resin of frankincense has been used in folk medicine and in religious ceremonies as a fumigant since time immemorial in the ancient world (Figure 1). In terms of botanical data, the genus *Boswellia* has 21 species, but the most popular ones are *Boswellia sacra*, *B. serrata*, *B. carterii*, and *B. papyrifera* [1]. Details of more than 15 other *Boswellia* species are illustrated in a conclusive book on the biology of the genus *Boswellia* [2]. Presently, there is a growing interest in the different biological activities of frankincense gum resin [3,4,5,6]. Among these biological activities, the anti-inflammatory and anticancer properties of the gum resin have caught the attention of many researchers. These properties originate from two main fractions of frankincense gum resin, namely the volatile fraction (essential oil) and the nonvolatile fraction. The following section will shed more light on both fractions and how the chemical constitution of each fraction is involved in the overall anti-inflammatory and anticancer properties of frankincense.

### 2.1. Volatile Fraction of Frankincense (the Essential Oil)

**Figure 2:**
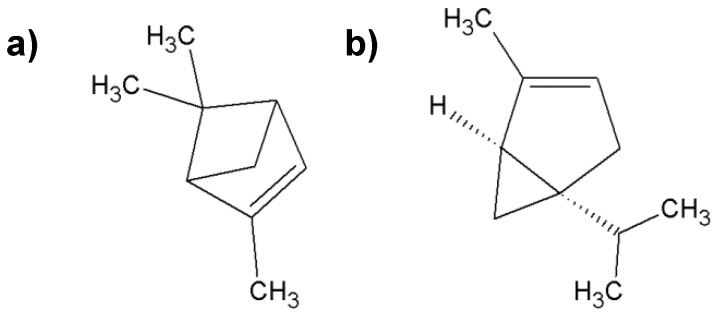
Characteristic compounds found in frankincense; (**a**) α-pinene; (**b**) α-thujene.

The volatile fraction is the fraction that can be isolated from the gum resin by using the traditional water or steam distillation method. This fraction is usually called frankincense essential oil (FEO) and forms 5%–15% of frankincense gum resin’s weight, depending on the species [1,2]. Presently, there are more developed environmentally friendly techniques, such as microwave-assisted distillation, that can be used to extract FEO [7]. The chemical composition of the essential oil was considered as a standard to differentiate between two important species of frankincense, namely *B. sacra* (native to the Arabian region) and *B. carterii* (native to Somalia). These two species were usually thought to be the same; however, Woolley et al. [8] proved that these two species are different based on the difference in the chemical composition of their essential oils. According to these authors, the essential oil of *B. sacra* is characterized by a high content of α-pinene (68.0%) and low content of α-thujene (0.6%). In contrast, the essential oil of *B. carterii* has a relatively lower content of α-pinene (37.0%) and higher content of α-thujene (7.9%) (Figure 2). Moreover, the chemical composition of FEO that belongs to *B. serrata* species (which is native to India) differs depending on the geographical region of the plant’s habitat [9].

Early studies have shown that FEO exhibits anticancer activity against breast and pancreatic cancer cell lines [10,11]. The antiproliferative and proapoptotic activities of FEO were also studied in cultured J82 (Malignant Human Urothelial Cell Line) uman bladder cancer cells and showed selective cancer cell death through NRF-2-mediated oxidative stress (nuclear factor (erythroid-derived 2)-like2) [12]. Hexane extraction of frankincense gum resin using the Soxhlet apparatus yielded the so-called heavy oil [13]. This heavy oil showed in vitro anticancer activity against breast cancer cells because of its constituents, including α-pinene (61.56%), α-amyrin (20.6%), and β-amyrin (8.1%). More recently, Hakkim et al. [14] showed that FEO suppresses melanoma cancer and ameliorates hepatotoxicity in vitro and in vivo. FEO also suppresses the cell viability, proliferation, migration, and invasion of the human breast cancer cell line MCF-7 [15]. In the field of cancer-supportive medicine, it was found that topical application of FEO can relieve cancer-related fatigue [16].

On the basis of the abovementioned findings, it can be concluded that the volatile fraction of frankincense (FEO) has a promising anticancer property. However, one should also note that there are other essential oils that have shown higher anticancer activity than FEO. For example, a recent study [17] showed that essential oils of cinnamon and *Litsea cubeba* are more active against different cancer cell lines than FEO. This result is not surprising to us because it is well known that the anticancer activity of frankincense gum [18] resin originates from the cumulative effect of its volatile fraction (essential oil) and the nonvolatile fraction. Therefore, the following section will shed more light on the role of the nonvolatile fraction in the overall anticancer activity of frankincense gum resin.

### 2.2. Nonvolatile Fraction of Frankincense (Boswellic Acids)

**Figure 3:**
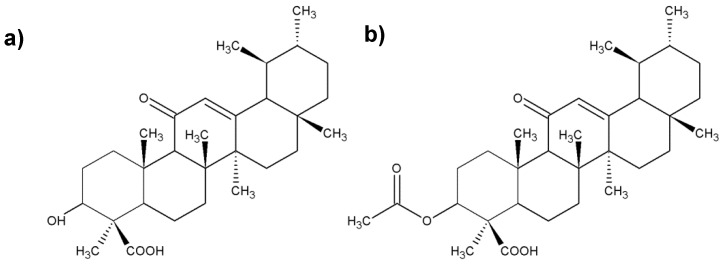

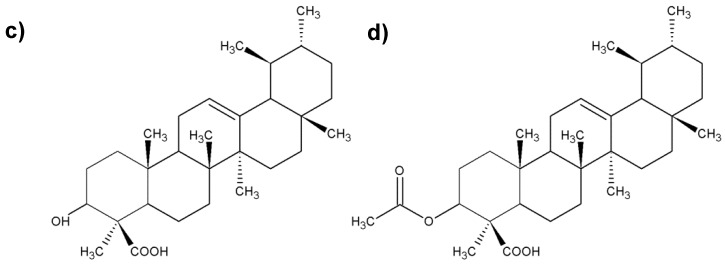
Chemical structure of the most important boswellic acids (BAs); (**a**) 3-O-acetyl-11-keto-β-boswellic acid; (**b**) 11-keto-β-boswellic; (**c**) acetyl-β-boswellic acid; (**d**) β-boswellic acid.

The nonvolatile fraction of frankincense is composed of pentacyclic triterpenic compounds known as boswellic acids (BAs). This group of nonvolatile molecules is generally produced by plants in the genus *Boswellia*. According to Buchele et al. [19], BAs mainly include four members: β-boswellic acid (β-BA), acetyl-β-boswellic acid (ABA), 11-keto-β-boswellic acid (KBA), and 3-O-acetyl-11-keto-β-boswellic acid (AKBA) (Figure 3).

In addition, two other BAs were identified, and all six BAs were analyzed and quantified using a developed chromatographic method [20]. It is important to note that there is a quantitative and qualitative difference in BAs among the different species of frankincense [2]. BAs are absent from the essential oil of frankincense because they are nonvolatile; therefore, they cannot be extracted by distillation. Alternatively, BAs can be extracted from the gum resin of frankincense by using other techniques. For instance, Niphadkar et al. [21] extracted one compound of the BA group (AKBA) from frankincense species (*B. serrata*) by using a technique called three-phase partitioning. Another technique based on ethanol-modified supercritical fluid extraction was also used to isolate the same BA member (AKBA) from frankincense gum resin [22]. It is worth noting that AKBA was reported to be the most anti-inflammatory and antitumor-active agent among the six BAs of frankincense [23]. The remaining members of the BA group were also reported to have anticancer properties [24]. For instance, β-BA showed anticancer properties against lung cancer by downregulation of the expression of survivin protein in addition to other mechanisms of action [25]. In addition, AKBA can reverse the multidrug resistance of ovarian cancer cells to therapeutic drugs, such as taxol, thereby making the cells more susceptible to chemotherapy. The mechanism of the anticancer activity of BAs mainly originates from the induction of apoptosis through caspase activation, increased Bax expression (modulation of pro-apoptotic), NF-κB (nuclear factor-κB) downregulation, and the induction of poly (ADP-ribose) polymerase cleavage [23]. Details of the chemistry of BAs and their mechanism of action against different tumor cells were reviewed in detail elsewhere [3,24,26]. Despite the significant anti-inflammatory and anticancer potential of BAs, their hydrophobic nature decreases their absorption, which renders them poorly bioavailable, especially for oral administration. Early studies revealed that the bioavailability of BAs can be enhanced after the administration of fatty meal [27,28]. Subsequently, the enhancement of the absorption of BAs was investigated using the lecithin delivery system known as phytosomes [29]. More recent studies used the micellar solubilization technique to enhance the absorption of BAs, which showed significant success [30]. Interestingly, this study showed that the increase in absorption depends on the type of each of the six BAs in the frankincense extract, and the highest absorption was detected with AKBA. Another approach to overcoming the low bioavailability of AKBA is by increasing its aqueous solubility through the formulation of inclusion complexes with β-cyclodextrin and hydroxypropyl-β-cyclodextrin [31]. On similar lines, cyclodextrin and poloxamer solid dispersion formulations were used to increase the intestinal absorption of BAs [32]. On the basis of the abovementioned findings, it can be concluded that the biological activity of frankincense, especially its anticancer properties, can be enhanced by a combination of its essential oil plus its boswellic acid content.

## 3. Biological Activity of Myrrh (*Commiphora myrrha*) Essential Oil

The biological activity of myrrh essential oil (MEO) has been studied to a lesser extent than that for FEO. This is apparent from the limited number of studies published to date on the biological activity of MEO. Therefore, the following text will review the few studies available concerning the biological properties of MEO.

**Figure 4:**
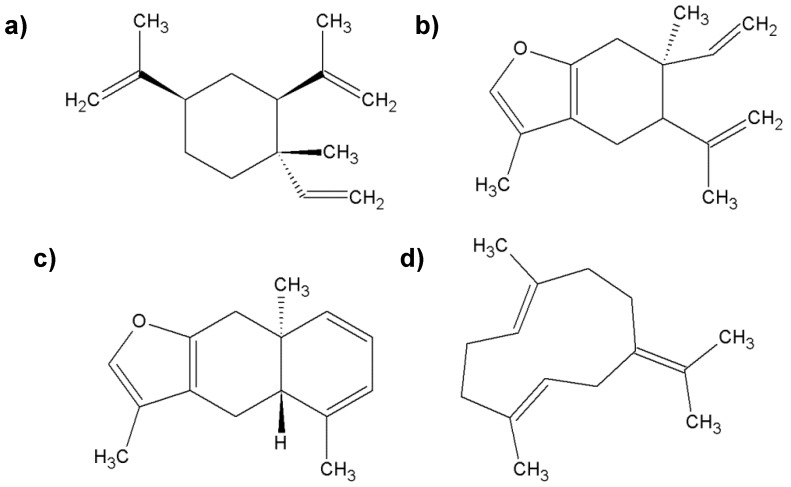
The most important chemical compounds found in myrrh; (**a**) β-elemene; (**b**) curzerene; (**c**) furanoeudesma-1,3-diene; (**d**) germacrene-B.

MEO is a volatile fraction isolated by distillation from the gum resins obtained from the plant *C. myrrha* (family Burseraceae). The oil has been used in folk medicine for a long time to treat different illnesses. The chemical composition of MEO differs radically from that of FEO, which can be taken as a standard for differentiating between these two gum resins. The gas chromatographic (GC) analysis of MEO indicates that the major volatile constituents are furanoeudesma-1,3-diene (17.65%), followed by curzerene (12.9%), β-elemene (12.7%), and germacrene-B (12.1%) [33] (Figure 4). On the other hand, a recent GC-mass spectroscopic study was performed on MEO and revealed that 2-acetoxy-furano-diene (26.82%) was the major volatile component of this essential oil [34].

MEO was subjected to a systematic study to reveal its biological activity. For example, a fraction of MEO contains some volatile sesquiterpene compounds that show anti-Alzheimer’s disease [35] and antiangiogenic effects [36]. In addition, MEO was found to be effective against some cancer cell lines, including liver, breast, and colon cancer cell lines. The same study also demonstrated high antimicrobial activity of MEO against some multidrug-resistant isolates [34]. The combination of MEO and sandalwood essential oils (Eos) showed a profound effect on pathogens that infect wounds [37]. MEO also showed profound antimicrobial activity against oral cavity pathogens, such as *Candida albicans*, which makes it a promising agent in oral health [38].

However, the ingestion of MEO without knowing its safe dose is considered to be hazardous due to its toxicity on the liver, kidney, and heart [39]. Therefore, acute and subacute toxicities of MEO were recently studied by subcutaneous injection in mice [40]. The results showed that low doses at 1, 5, and 10 μL MEO/mice are safe for administration, while higher doses are toxic and cause damage to different body organs. Unfortunately, no other substantial studies on the activity of MEO were found. However, some other studies that investigated the activity of the ethanolic or aqueous extract of the whole gum resin of myrrh are available, [41,42]. In these investigations, other bioactive components detected in myrrh (rather than the essential oil per se) were found to be responsible for the biological activity.

## 4. Biological Activity of Ginger (*Zingiber officinale*)

**Figure 5:**
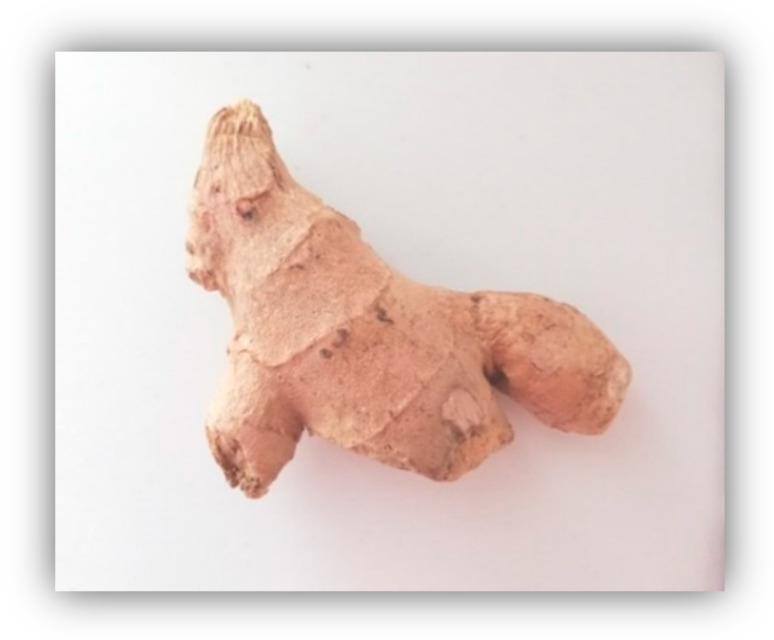
Raw ginger root.

Ginger is a plant from the ginger family (Zingiberaceae) (Figure 5) [43]. It produces a stem (60–90 cm long) covered with dark green leaves. It owes its name to the English botanist William Roscoe. The most important parts of this plant, from a health and food point of view, are underground rhizomes divided into tuberous sections. Rhizomes are light brown and covered with a thick wrinkled bark [44,45]. Ginger grows in a warm humid climate. It is grown mainly at an altitude of 1500 m above sea level. Soil for ginger cultivation should be well drained and rich in organic matter (soil pH should be 6.0–6.5). Ginger is grown mainly in China, Nepal, India, Taiwan, Bangladesh, and Nigeria. India is the largest producer of this plant [43,46,47].

Ginger is a spice used in food and drinks because of its characteristic pungency and spicy taste; in addition, it is an excellent source of many biologically active compounds, including bioactive phenols (nonvolatile sharp compounds, such as gingerols, parades, shogaols, and zingerones). In traditional medicine, ginger has been used since antiquity (>2500 years). This spice is known for increasing gastrointestinal motility and has antibacterial, antiviral, analgesic, and antipyretic properties. Scientific reports indicate that ginger also has anti-inflammatory, anticancer, antihyperglycemic, and antilipidemic properties. Scientific research clearly indicates that because of its health-promoting effects, it is worthwhile to include ginger in your daily diet [48].

Ginger contains many volatile oils, oleoresins, and macroelements (phosphorus, magnesium, and potassium). Ginger root is a valuable raw material containing bioactive substances with antioxidant and anti-inflammatory effects. The composition of ginger depends mainly on the place of origin of the plant and the condition of the rhizomes (fresh or dried). The edible part of ginger contains basic nutrients: Proteins, fats, carbohydrates, dietary fibers, vitamins, and minerals [49,50]. Ginger rhizome contains 60%–70% carbohydrates (the majority is starch), 3%–8% crude fiber, 9% protein, 8% ash, and 6% fatty oil. Among lipids, free fatty acids (e.g., palmitic, oleic, linoleic, caprylic, capric, lauric, myristic, pentadecan, heptadecan, stearic, linolenic, and peanut), lecithin, phosphatidic acid, and glycolipids are found. Amino acids found in ginger are arginine, aspartic acid, cysteine, glycine, isoleucine, leucine, serine, threonine, and valine [43].

**Figure 6:**
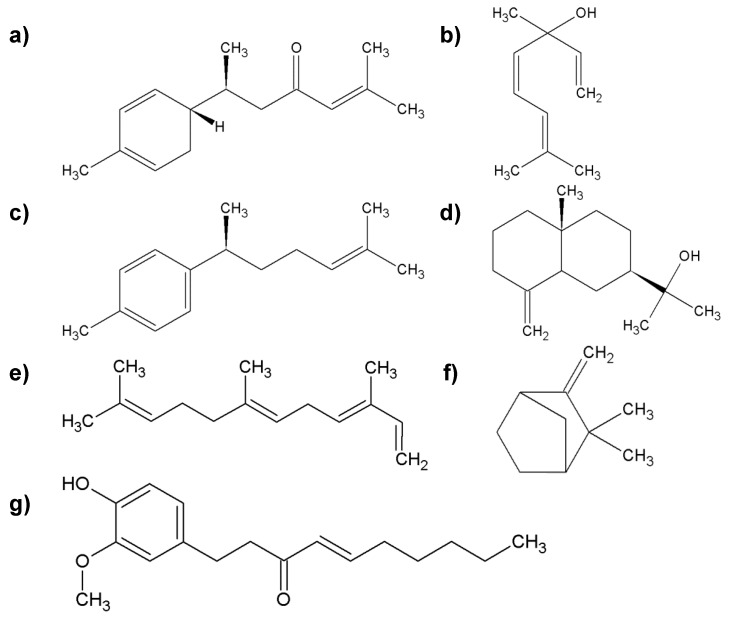
The most important group of chemicals found in ginger root; (**a**) α-zingiberene; (**b**) linalool; (**c**) ar-curcumene; (**d**) zingiberol; (**e**) α-farnezene; (**f**) camphene; (**g**) shogaol.

Fresh ginger is more effective in combating diseases and other illnesses than dried ginger. The health properties of ginger are mainly influenced by phytonutrients, which include volatile and nonvolatile compounds. The first group consists of monoterpenes and sesquiterpenes: α-zingiberene, zingiberol, α-farnezene, β-bisabolen, camphene, cineol, linalool, limonene, geraniol, terpineol, and ar-curcumene (Figure 6). The second group consists of zingerone, gingerols (e.g., 6-gingerol and 10-gingerol; they are responsible for the pungency of fresh ginger), and shogaol (determines the pungency of dried ginger), which originate from the dehydration of gingerols [51,52].

Literature data suggest that this spice was a highly valued commodity as early as the 13th and 14th centuries [53]. Ginger was used (and is still being used) in traditional Chinese medicine to treat, for example, indigestion, flatulence, nausea, ulcers, headache, arthritis, and pneumonia [46]. Underground rhizomes are processed into powder, syrup, oils, and oleoresin. Ginger has GRAS status (Generally Recognized as Safe), and hence, it has a wide spectrum of applications. It is used, for example, for the production of dietary supplements and as an additive to beverages (e.g., beer) or food products (curry, soups, jams, bread, and confectionery) [54]. Most clinical trials used between 250 mg and 1 g of powdered root in a capsule form (up to four times a day). Marx et al. [55] suggested that a typical dose of ginger to prevent nausea and vomiting should be in the range of 1 g of powdered dry rhizome (i.e., 2 to 4 g of fresh rhizome). For the treatment of inflammation, it is recommended to consume up to 3 g of powdered ginger per day. Despite its health-promoting properties, ginger cannot be consumed by everyone, including breastfeeding women, because of its pungency. In addition, it should not be consumed by people prone to heartburn, those suffering from stomach ulcers or duodenal ulcers, and those awaiting hospital procedures [43].

Ginger stimulates the production of saliva and bile acid. Prakash and Srinivasan [56] observed that ginger intensifies the digestion and absorption of fats in a high-fat diet due to more effective bile acid secretion by the liver and higher pancreatic lipase activity. According to Platel and Srinivasan [57,58], ginger stimulates the activity of amylase, trypsin, chymotrypsin, and carboxypeptidase. Wang et al. [59] observed a decrease in body weight and liver steatosis, low-grade inflammation, and amelioration of insulin resistance in ginger-treated mice on a high-fat diet. Moreover, supplementation with ginger caused modulation of the gut microbiota composition and increased bacteria of the genus *Bifidobacterium* and bacteria producing short-chain fatty acids (*Alloprevotella* and *Allobaculum*). An increase in the concentration of short-chain fatty acids in feces was also detected.

In ginger-fed rats, the intestinal brush border membrane (BBM) showed increased fluidity and permeability, resulting in an increase in the absorbent surface of the small intestine. This was accompanied by a reduced ratio of cholesterol: phospholipids in the jejunum and ileum of the small intestine and an increase in the activity of the enzymes of BBM, namely glycyl glycine dipeptidase, leucine aminopeptidase, and gamma-glutamyl transpeptidase, in the small intestinal mucosa. Ginger-fed animals were shown to have more efficient intestinal absorption of iron, zinc, calcium, and beta-carotene. The beneficial effect of ginger on mucosal glycoproteins has also been described, and ginger has been shown to confer protective effects on the gastrointestinal tract [60,61].

Several studies have reported the effectiveness of ginger in suppressing cholesterol and lipid accumulation in mammals. ElRokh et al. [62] proved that ginger intake improves the blood lipid profile. They administered rats with confirmed hypercholesterolemia (caused by a high-fat diet) an aqueous ginger extract as a diet supplement for 4 weeks. At the end of the experiment, a decrease in the total cholesterol level was observed in the blood. Another study showed that the level of cholesterol reduction was higher in the group supplemented with ginger extract than in the group administered atorvastatin (a hypolipemic drug). A hypolipemic effect was also found in diabetic mice. For 12 days, they were given 6-gingerol isolated from ginger (100 mg/kg body weight). This diet decreased triglyceride levels (by 41.1%), total cholesterol (by 31.2%), and low-density lipoprotein (LDL) cholesterol (by 27.9%). The concentration of free fatty acids also decreased (by 24.4%) [63]. Ginger’s lipid-lowering properties were confirmed by Taha et al. [64]. They administered hyperlipidemic rats with meals containing ginger extract at a dose of 400 mg/kg body weight. A decrease in total cholesterol, triglycerides, and LDL cholesterol was found. Previous studies have confirmed the effect of aqueous ginger extract on serum cholesterol and triglyceride levels as well as on the production of platelet thromboxane and prostaglandin E2 in rats [65]. Ginger appears to be effective in reducing cholesterol and lipid buildup in the body; thus, it can help to control body weight. Ginger has been shown to regulate the PPARδ (peroxisome proliferator-activated receptor δ) signaling pathway in adipocytes and reduce the risk of obesity in C57BL/6J mice [66]. 6-Shogaol and 6-gingerol stimulated PPARδ-dependent gene expression in cultured human skeletal muscle myotubes. Current research suggests that ginger extract (containing 6-shogaol and 6-gingerol) alleviates diet-induced obesity and improves the exercise capacity of the body by increasing fat catabolism in skeletal muscle due to the activation of the PPARδ pathway. Gingerol prevents hyperlipidemia by modulating the expression of enzymes involved in the maintenance of cholesterol homeostasis [31]. Ginger extract showed cardioprotective potential and prevented isoproterenol-induced experimental myocardial infarction in Wistar rats [67].

Some scientific reports suggest the hypoglycemic properties of ginger. Oludoyin and Adegoke [68] conducted a study in which diabetic-induced rats were given an extract of fresh or cooked ginger for 4 weeks (4 cm^3^/kg body weight). Both types of extracts were shown to lower sugar levels to pre-disease levels. Similar results were reported by Jafri et al. [69]. They used ginger extract at a dose of 500 mg/kg body weight. Kondeti Ramudu et al. [70] confirmed these reports. Ginger exerts a hypoglycemic effect through the following mechanisms: Increased tissue sensitivity to insulin, stimulation of insulin release, reduction of glucose absorption from the intestine, and inhibition of digestive enzymes, mainly pancreatic amylase [71]. Gingerols and shogaol contained in ginger are responsible for preventing diabetes. Ginger has also been shown to have a significant protective effect on diabetic complications, including complications related to the liver, kidneys, eyes, and nervous system [72].

Excessive production of free radicals that exceeds their removal rate leads to oxidative stress, which plays a critical role in the development of degenerative diseases. The antioxidant activity of ginger includes, among others, scavenging free radicals, suppressing lipid peroxidation, strengthening antioxidant molecules, stimulating endogenous antioxidant enzymes, and inhibiting LDL oxidation or arachidonate metabolizing enzymes: 5-lipoxygenase and 2-cyclooxygenase [73]. The total antioxidant capacity of ginger was determined to be based on the content of flavonoid, phenolic acid, and polyphenols [74]. The obtained results showed a positive linear correlation between the total antioxidant capacity and the natural phenolic compounds contained in ginger. The study proved that ginger rhizomes are a potential source of natural antioxidants, mainly 6-gingerol and 6-paradol [75]. 6-Gingerol is a strong inhibitor of nitric oxide (NO) synthesis and a protective factor for macrophages against peroxynitrite-mediated damage [76]. Reduced levels of lipid peroxide were found in ginger-fed diabetic rats. The results obtained by Shanmugam et al. [77] suggest that ginger has a neuroprotective effect by accelerating the defense mechanisms of antioxidants in the brain. Attia et al. [78] found that diabetic rats receiving ginger were characterized by a lower concentration of malonic dialdehyde and a higher level of antioxidant enzymes, namely superoxide dismutase, glutathione peroxidase, and glutathione, than in animals given a ginger-deficient diet. Hinneburg et al. [79] showed that aqueous ginger extracts can reduce iron (III) ions to iron (II) ions and scavenge 1,1-diphenyl-2-picrylhydrazyl (DPPH) free radicals.

In vitro studies and animal experiments indicate the anti-inflammatory potential of ginger. Lipid peroxides and activated macrophages play an important role in inflammation (e.g., joints). Ginger ingredients inhibit the inflammatory process by inhibiting arachidonic acid metabolism [80]. Studies have shown that ginger and its components inhibit both cyclooxygenase and lipoxygenase, and limit leukotriene synthesis [81]. Hsiang et al. [82] suggest that ginger and zingerone have anti-inflammatory effects as they inhibit NF-kB activation, Interleukin-1β(IL-1β) production, and the infiltration of inflammatory cells (ginger and zingerone suppress lipopolysaccharide (LPS)-induced systemic inflammation by cytokine production). Lantz et al. [83] proved the anti-inflammatory activity of 6-, 8-, and 10-gingerol and 6-shogaol (from ginger rhizome).

It is possible that ginger has a positive effect of cancer prevention. This property is attributed to the individual components of this spice, mainly 6-gingerol, 6-paradol, and zingerone. The probable mechanisms associated with the preventive potential of ginger and its ingredients have been proven in laboratory animal tests by using several experimental models. There is increasing evidence that ginger and its ingredients inhibit the development of various types of cancer cells. The antitumor potential of ginger has been studied in many types of cancer (skin, prostate, pancreatic, colorectal, and blood) using cancer cell lines or animal models [84,85,86,87]. Karna et al. [88] found that daily oral administration of ginger extract inhibits the growth of human prostate cancer cells, while Akimoto et al. [89] showed that ethanolic extract of ginger induces the death of human pancreatic cancer cells. Experimental studies have proved that ginger and its biologically active ingredients (6-gingerol and 6-shogaol) show anticancer activity against gastrointestinal cancer. The chemopreventive role of these compounds is associated with their ability to modulate signaling molecules, such as NF-kB, tumor necrosis factor-α (TNF-α), cyclooxygenase-2 (COX-2), B cell CLL/lymphoma-2 (Bcl-2), and caspases, and other proteins that regulate cell growth [90]. According to Radhakrishnan et al. [91], the sensitivity of colon cancer cells to 6-gingerol is associated with the activation of caspases (8, 9, 3, and 7), suggesting the induction of cell apoptosis. Inhibition of the ERK1/2 (extracellular signaling kinases)/JNK (c-Jun N-terminal kinase)/AP-1 (activator protein-1) pathway is considered to be a possible mode of action for 6-gingerol [92]. Zerumbone is another ingredient of ginger with an anticancer effect. In the study of the effect of zerumbone on angiogenesis associated with pancreatic cancer, this compound inhibited mRNA expression and protein secretion by angiogenic factors and NF-kB (angiogenesis is essential for tumor growth and metastasis). Karna et al. [88] found that ginger causes mitochondrial apoptosis of human prostate cancer cells; furthermore, it disturbs cell cycle progression and modulation, affects reproductive capacity, and destroys regulatory molecules.

Ginger has also been reported to have a protective effect on kidneys exposed to alcohol and to exert hepatoprotective effects [93]. 6-Gingerol has been tested for antiallergic effects [94], and it reduced the severity of allergic symptoms in mice with allergic rhinitis caused by ovalbumin. Menstrual pains affect most menstruating women, making their everyday life more difficult. Prostaglandins are responsible for this effect. Ginger components inhibit cyclooxygenase and lipoxygenase, which are the enzymes involved in the synthesis of prostaglandins [95]. Literature data also suggest antimigraine effects of ginger [96].

## 5. Biological Activity of Turmeric (*Curcuma* sp.)

Plant species belonging to the genus *Curcuma* are widely used as spices in food and folk medicine. *Curcuma* is included in the Zingiberaceae family, which includes 52 genera and over 1300 species [97]. Plants belonging to this family naturally occur in Southeast Asia, New Guinea, and northern Australia [98]. Currently, 93 to 100 species are included in the genus *Curcuma*, but this classification changes frequently. Plants belonging to this genus are commonly used as a source of dyes and spices in traditional Asian cuisine. *Curcuma* is commonly used to treat pneumonia, bronchial diseases, diarrhea, and purulent wounds. For this purpose, the rhizome of the plant is used, which is a rich source of active ingredients, among which curcuminoids have a special effect [99]. Of the dozens of species included in the genus *Curcuma*, curcuminoids have been isolated from *C. aromatica*, *C. comosa*, *C. longa*, *C. mangga*, *C. phaeocaulis*, *C. xanthorrhiza*, and C. *zedoaria* [100]. The following text describes the properties of two *Curcuma* species: *C. longa* (turmeric) and *C. zedoaria* (zedoary), because they are most often grown and used commercially worldwide [99].

### 5.1. Curcuma Longa (l.)

**Figure 7:**
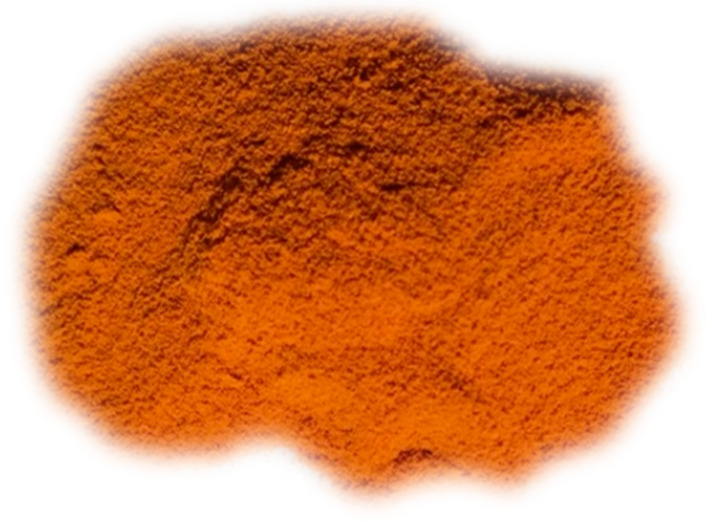
Turmeric powder.

*Curcuma longa* (l.) is commonly referred to as turmeric worldwide (Figure 7). In Arabic, the term “kurkum” is also used, in Hindi “haldi” [101], in Chinese “Chiang Huang”, and in Japanese “Ukon” [102]. It is widely grown in India, China, Pakistan, Ghana, Kenya, and Nigeria, which makes its rhizomes easily available and cheap [103]. It is a perennial plant with short stems. It can grow up to 100 cm tall. The leaves are ovate, curved, and oblong, and the rhizomes are cylindrical. Flowers can be white or colorful [101]. The first mention of turmeric can be found in Marco Polo’s writings about his trip to China and India from 1280. Turmeric was first introduced to the European market by Arab traders in the 13th century. In the following years, the British colonizing India imported turmeric to Europe under the name “curry powder” [100].

**Figure 8:**
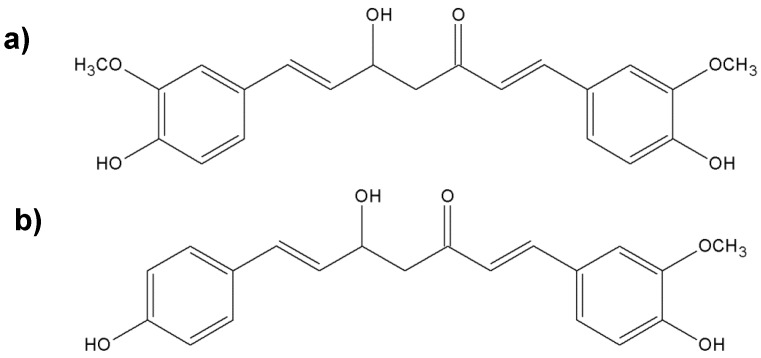

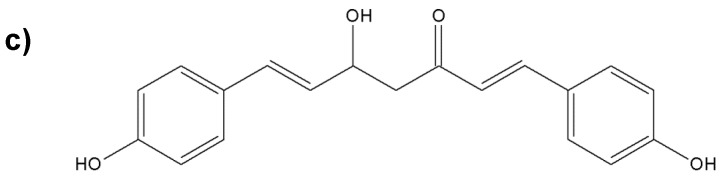
Chemical structures of the nonvolatile important components of turmeric; (**a**) diferuloylmethane; (**b**) desmethoxycurcumin; (**c**) bisdesmethoxycurcumin.

Currently, turmeric and its biologically active ingredients are intensively studied worldwide. Turmeric has been found to have anti-inflammatory [104], antioxidant [105], anticancer [106], antidiabetic [107], and antimalarial activities [108]. In traditional medicine, this plant is used as a laxative, carminative, and digestive aid. It is also used to treat fever, gastritis, food poisoning, respiratory tract infections, hypercholesterolemia, hypertension, jaundice, urinary tract infections, rheumatoid arthritis, and skin diseases and to relieve menstrual pain [99,109]. Turmeric is classified by the US Food and Drug Administration (US FDA) as a nutraceutical that is considered to be safe and has GRAS status [103]. The yellow color of turmeric is due to the presence of curcuminoids [110], which usually constitute 3%–5% of turmeric preparations. Turmeric alcohol extract contains three main curcumins, i.e., turmeric (also known as curcumin I or diferuloylmethane), desmethoxycurcumin (curcumin II), and bisdesmethoxycurcumin (curcumin III). Most commercial preparations of turmeric found under the name “curcumin” are a mixture of curcumin (approximately 77%), desmethoxycurcumin (approximately 18%), and bisdesmethoxycurcumin (approximately 5%) [102] (Figure 8).

**Figure 9:**
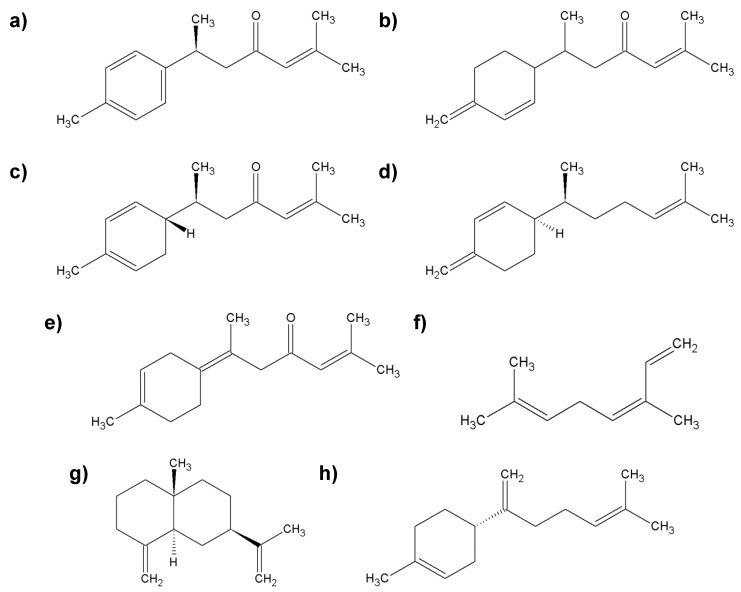
Characteristic compounds found in turmeric oil; (**a**) *ar*-turmerone; (**b**) β-turmerone; (**c**) α-zingiberene; (**d**) β-sesquiphellandrene; (**e**) (*E*)-γ-atlantone; (**f**) (*Z*)-β-ocimene; (**g**) β-selinene; (**h**) β-bisabolene.

Turmeric oil is one of the forms of *C. longa* (l.) that is rich in active ingredients. On an industrial scale, it is isolated from a byproduct after the extraction of curcumin, commonly known as “cumin-removed turmeric oleoresin”. Turmeric oil is extracted from the oleoresin, usually with hexane. It is a product with varying composition, which results from the genotype of the plant, place of cultivation, weather conditions, season of the year, fertilizers used, methods of cultivation and storage after harvest, and extraction methods [99]. The main active ingredients of turmeric oil are essential oils and curcuminoids. Hundreds of chemicals have been identified in turmeric oil, including *ar*-turmerone, α-turmerone, β-turmerone, α-zingiberene, ar-curcumene, β-sesquiphellandrene, β-caryophyllene, (*Z*)-β-ocimene, β-bisabolene, (*Z*)-β-farnesene, β-caryophyllene, humulene oxide, α-phellandrene, caryophyllene oxide, β-selinene, (E)-γ-atlantone, terpinolene, 1,8-cineole, and caryophyllene oxide [111] (Figure 9).

Liju et al. [112] tested the antioxidant and anti-inflammatory activity of the essential oils of *C. longa* (l.), whose main ingredients were ar-turmerone (61.79%), curlone (12.48%), and ar-curcumene (6.11%). In the in vitro study, turmeric oil showed antioxidant activity, and the ability to capture hydroxyl radicals. The authors also found that feeding mice with turmeric oil for 1 month increased the levels of superoxide dismutase, glutathione, and glutathione reductase in the blood of the animals. The levels of glutathione S-transferase and superoxide dismutase in the liver also increased.

Turmeric oils can inhibit the growth of various pathogenic bacteria, including Gram-negative species, such as *Helicobacter pylori*, *Proteus mirabilis*, *P. aeruginosa*, *E. coli*, and *Vibrio parahaemolyticus*, and Gram-positive species, such as *Bacillus cereus*, *B. coagulans*, and *S. aureus*. Strong antifungal activity was also found against *A. niger* and *A. flavus*, *Cochliobolus miyabeanus*, *Cochliobolus lunatus*, *Fusarium verticillioides* and *F. oxysporum*, *Penicillium digitatum*, *Alternaria dianthi*, and *Colletotrichum falcatum* [113,114,115,116]. In addition, oils obtained from turmeric significantly affected the metabolism of the mold *A. flavus*, thereby interfering with the synthesis of aflatoxins. Ferreira et al. [101] found that turmeric oil at a concentration of 0.5% showed the best inhibitory effect (96%).

### 5.2. Curcuma Zedoaria (Christm.)

*Curcuma zedoaria* is known worldwide as “zedoary” and “white turmeric”. The natural habitat of this plant is in India and Indonesia, but because of its ease of cultivation, it is also grown in countries, such as Thailand, Japan, and China [99,117]. The rhizomes of zedoary resemble those of ginger, and after cutting, they have a color similar to that of turmeric. They have a less intense aroma, which is rated as turmeric mixed with mangoes, and therefore, powdered rhizomes are used for culinary purposes [99]. Zedoary in the form of oil, juice, or fresh rhizomes has been used for centuries in traditional medicine in India, especially for treating stomach pain, vomiting, and food poisoning; for reducing menstrual pain; and for eliminating parasites in children [117,118]. Essential oils obtained from the rhizomes of this plant are a rich source of active ingredients [119]. The biologically active ingredients include epicurzerene, curzerene, curdione, curzerenone, debromofiliforminol, 1,8-cineol, β-sesquiphellandrene, p-cymene, curcumenene, and α-phellandrene [99]. Zedoary essential oils can be used as natural antimicrobial agents because they show activity against Gram-negative bacteria (*Salmonella* Typhimurium, *V. parahaemolyticus*, *P. aeruginosa,* and *E. coli*), Gram-positive bacteria (*S. aureus*, *B. cereus*, and *Corynebacterium amycolatum*), and fungi (*Colletotrichum falcatum*, *Phytophthora capsici*, *Candida albicans*, and *Aspergillus ochraceus*) [120,121,122]. It was also found that zedoary oil at a concentration of 20 mg/L had strong antioxidant properties and an excellent ability to scavenge DPPH free radicals [123].

### 5.3. Anti-Inflammatory Effect of Curcumin and Curcuminoids

Curcumin is one of the polyphenolic compounds, and the molecule is composed of two phenyl rings substituted with methoxy and hydroxyl groups, which are linked by a seven-carbon ketoenol linkage [124]. Under physiological conditions, curcumin is in equilibrium with two enol and bis-keto forms [125]. As a safe coloring agent, curcumin is approved for use as a food additive under number E100. It is recognized as safe by the Joint Expert Committee of the Food and Agriculture Organization/World Health Organization (FAO/WHO) [102]. Curcuminoids have antioxidant and anti-inflammatory properties. In 2015, the Sahebkar team evaluated the effectiveness of curcuminoid supplementation on the activity of superoxide dismutase (SOD), catalase (CAT), and glutathione (GSH) and lipid peroxides as parameters of oxidative stress by meta-analysis of randomized data. During curcuminoid supplementation, a significant increase was observed in SOD activity (weighted mean difference, WMD = 1.15 U/mL) and a significant increase in SOD activity occurred after 6 weeks of supplementation (WMD = 1.46 U/mL). Curcuminoids significantly reduced the level of lipid oxidation products in blood serum (WMD = −6.35 nmol/mL), while the level of GSH (WMD = 5.39 µg/mL) and CAT activity (WMD = 51.78 U/mL) increased. These properties of curcuminoids mean that turmeric is considered as a very effective antioxidant compound [126].

Because of its anti-inflammatory properties, curcuminoids can be considered as an alternative for the treatment of rheumatoid arthritis [127]. Turmeric has been shown to increase flexibility and reduce joint swelling in people with rheumatoid arthritis. An in vivo study by Wang et al. [128] showed that curcumin reduced the degree of swelling in the joints of rats. Curcumin was also shown to exert a therapeutic effect on arthritis in CIA rats and has a strong pharmacological effect on reducing the inflammatory response in macrophages. It is worth noting that curcumin has been shown to be more effective in relieving the symptoms of rheumatoid arthritis, such as tenderness and swelling in the joints, than conventional medicines. However, its usefulness as a therapeutic agent is limited by poor absorption and rapid metabolism [129].

Curcuminoids have anticancer properties. The first mechanism involves inhibiting arachidonic acid metabolism [130] by partially inhibiting the formation of prostaglandins (PGs). Numerous scientific studies have confirmed that PGs are one of the groups of mediators that stimulate cell proliferation, including cells of various types of cancer. The key enzyme responsible for the conversion of arachidonic acid is cyclooxygenase (COX), which occurs in the form of two isoforms designated as COX-1 and COX-2. The COX-1 form is present in almost all tissues and is involved in maintaining homeostasis. Inhibition of its activity causes serious health effects, such as impaired renal blood flow. COX-2 is activated during the inflammatory reaction when the immune response develops, and its overexpression leads to cancerogenesis of the lung, breast, pancreas, prostate, colon, and rectum [131]. Goel et al. [132] conducted a study to determine the effect of curcumin on COX-2 expression. They used the HT-29 (ATCC ^®^ HTB-38 ™, Manassas, VA, USA) human colon cancer cell line and found that curcumin inhibited HT-29 cell growth in a concentration- and time-dependent manner. The maximum inhibitory effect was found at 75 mM curcumin after 72 h. Curcumin significantly inhibited COX-2 mRNA and protein expression but did not affect COX-1 activity. Zhang et al. [133] conducted research on the effect of curcumin on the activity of two known tumor promoters, namely bile acids (BA) and forbol esters (PMA), on human gastrointestinal cancer cell lines (SK-GT-4, IEC-18 (ATCC^®^ CRL-1589™), and HCA-7 (ECACC General Cell Collection, Salisbury, UK). Studies have shown that the presence of curcumin inhibited BA- and PMA-induced COX-2 induction and E_2_ prostaglandin synthesis.

The second mechanism relates to the inhibition of nitric oxide synthase (iNOS) activity. This enzyme catalyzes the oxidative deamination reaction of L-arginine, during which nitric oxide is produced. It has strong inflammatory, mutagenic, and carcinogenic effects. It affects tumor development by regulating angiogenesis, probably by stimulating the production of vascular endothelial growth factor (VEGF). Nitric oxide reacts with a superoxide anion, and the peroxynitrite (ONOO-) thus formed in this reaction can cause DNA damage. Chan et al. [134] tested the effect of curcumin in an experiment conducted on adult BALB/c mice. The authors showed that the oral administration of 0.5 mL of a 10 mM curcumin solution (92 ng/g body weight) reduced NOS activity in the liver of mice injected with LPS by 50%–70%.

The third mechanism concerns the reduced expression of various inflammatory cytokines, which leads to an inhibition of tumor growth [130]. Cho et al. [135] conducted research on the effect of curcumin on the expression of proinflammatory cytokines and cyclin E in cells of the HaCaT cell line treated with tumor necrosis factor (TNF). The authors found that the expression of IL-1β, IL-6, and TNF-α cytokines, however, did not affect the expression of IL-8 cytokines. This activity was associated with inhibition of the NF-κB and MAPK pathways.

## 6. Conclusions

It is clear from the abovementioned results obtained using animal models, cell lines, and other in vitro assessments that the four aromatic plants included in this review can seriously be considered as potential natural pharmaceutical resources. The combination of volatile and nonvolatile fractions of these plants can increase the biological activity. Therefore, we suggest consideration of these extracts for preclinical evaluation for possible future application as adjuvants to classical synthetic drugs for more profound protective or therapeutic activity. The challenge that can confront this trend is the standardization of the plant extracts so that they contain reproducible concentrations of the bioactive compounds. This is an important issue because plant bioactives are secondary metabolites that are affected by different factors, such as the geographical origin, plant species, and other agricultural practices used for the growth of the aromatic plant. It is worth emphasizing that these plants are being actively studied by various research groups in the world. The skillful use of plant wealth will allow us to avoid various unexpected medical conditions in the future. Their use will strengthen the body’s resistance and enable other sources of the use of these plant resources to be sought. This article supplements and extends the existing knowledge on these plants.
